# Multistep 11-*cis* to All-*trans* Retinal Photoisomerization in Bestrhodopsin, an Unusual Microbial Rhodopsin

**DOI:** 10.1021/jacs.5c06216

**Published:** 2025-07-10

**Authors:** Matthias Broser, Spyridon Kaziannis, Ivo H.M. van Stokkum, Atripan Mukherjee, Jakub Dostal, Wayne Busse, Arno Munhoven, Cesar Bernardo, Peter Hegemann, Miroslav Kloz, John T.M. Kennis

**Affiliations:** † Institut für Biologie, Experimentelle Biophysik, 9373 Humboldt Universität zu Berlin, Invalidenstrasse 42, Berlin D-10115, Germany; ‡ ELI Beamlines Facility, 1190 The Extreme Light Infrastructure ERIC, Za Radnicí 835, Dolní Břežany 252 41, Czech Republic; § Department of Physics, University of Ioannina, Ioannina Gr-45110, Greece; ∥ Department of Physics and Astronomy, 159203 Vrije Universiteit Amsterdam, De Boelelaan 1100, Amsterdam 1081 HV, the Netherlands

## Abstract

Rhodopsins constitute a broad class of retinal-binding photoreceptors. Microbial rhodopsins are canonically activated through an all-*trans* to 13-*cis* photoisomerization, whereas animal rhodopsins are mostly activated through an 11-*cis* to all-*trans* isomerization. Bestrhodopsins constitute a special microbial rhodopsin subfamily, with bistable rhodopsin domains that can be photoswitched between a far red-absorbing state D661 and a green-absorbing state P540. Its photochemistry involves a peculiar all*-trans* to *11-*cis** isomerization for the D661 to P540 photoreaction and vice versa. Here, we present the bestrhodopsin 11-*cis* to all-*trans* photoreaction as determined by femtosecond-to-submillisecond transient absorption, femtosecond stimulated Raman and flash-photolysis spectroscopy. The primary photoreaction involves ultrafast isomerizations in 240 fs from the 11-*cis* reactant to a mixture of highly distorted all-*trans* and 13-*cis* photoproducts. The 13-*cis* fraction then thermally isomerizes to a distorted all-*trans* RSB in 120 ps. We propose bicycle pedal models for the branched photoisomerizations with corotation of the C11=C12 and C13=C14 double bonds. One reactant fraction undergoes bicycle pedal motion aborted at the C13=C14 double bond, resulting in all-*trans* retinal. The other fraction undergoes a full bicycle pedal motion of both C11=C12 and C13=C14, resulting in 13-*cis* retinal. The primary products are trapped high up the ground-state potential energy surface with a low energetic barrier that facilitates thermal isomerization from 13-*cis* to all-*trans* retinal in 120 ps. All-*trans* retinal then structurally and energetically relaxes with subsequent time constants of 0.7 and 62 μs and 4.4 ms, along with counterion protonation, completing the P540 to D661 photoreaction.

## Graphical abstract

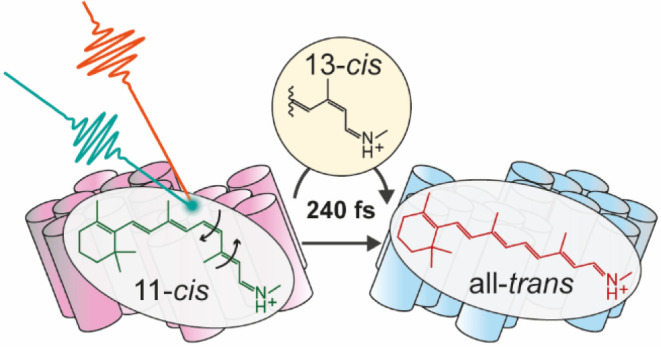

## Introduction

Rhodopsins constitute a broad class of sensory photoreceptors with retinal chromophores bound to the protein via a retinal Schiff base (RSB). They are classified as Type I and Type II, where the former are found in microbes and the latter in animals.^1^ Microbial rhodopsins are mostly activated through an all-*trans* to 13-*cis* photoisomerization reaction of the RSB, whereas animal rhodopsins are predominantly activated through an 11-*cis* to all *trans* isomerization reaction.^1^ The photoisomerization reactions occur on the ultrafast time scale and have been studied by time-resolved electronic^2–10^ and vibrational spectroscopy,^11–16^ X-ray crystallography,^17–20^ and quantum chemical computational methods.^21,22^

The ultrafast 11-*cis* to all-*trans* isomerization reaction has been extensively studied for bovine rhodopsin and to some extent for squid rhodopsin,^23,24^ exemplifying an iconic photoreaction with an exceedingly fast ballistic,^3,5^ vibrationally coherent photoisomerization,^2,4,25,26^ a direct curve crossing that is complete in less than 200 fs at an efficiency of 65%.^21^ The isomerization time scales and nature for the microbial rhodopsins all-*trans* to 13-*cis* reaction vary considerably, with some systems such as bacteriorhodopsin, channelrhodopsin and cation pumps reacting nearly barrierless in a few hundreds of fs^7–9,14,16,27–30^ and in other systems diffusive motions on the excited-state potential energy surface (PES) take place before isomerization or recombination takes place on the subps to ps time scale.^8,10,31,32^ with variable quantum quantum yields between 10–60%.^28,33–35^

The recently discovered bestrhodopsins constitute a special rhodopsin subfamily the members of which consist of one rhodopsin or two fused rhodopsins in tandem and a bestrophin ion channel domain, forming large pentameric structures with 5 or 10 rhodopsin modules.^36^ The rhodopsin domains of bestrhodopsin (PaRR), used in our study, comprise an all*-trans* RSB with unusually red-shifted absorption peaking near 660 nm,^34^ which is the reddest absorption for any native rhodopsin except for neorhodopsin.^37–42^ In addition, the bestrhodopsin photochemistry involves a peculiar all*-trans* to *11-*cis** isomerization^34,36^ rather than the all*-trans* to *13-*cis** photoreaction of canonical microbial rhodopsins.^1^ PaRR is bistable and can be photoswitched back and forth between the red-absorbing dark state D661, with an all-*trans* RSB and the green-absorbing assumed active state P540, with an 11-*cis* RSB (Figure 1). We recently characterized the D661 to P540 forward reaction, and found an unusual and complex reaction cascade involving multiple isomerizations involving 11-*cis* and 13-*cis* RSB on the ground state potential energy surface (PES).^34^

**Figure 1:**
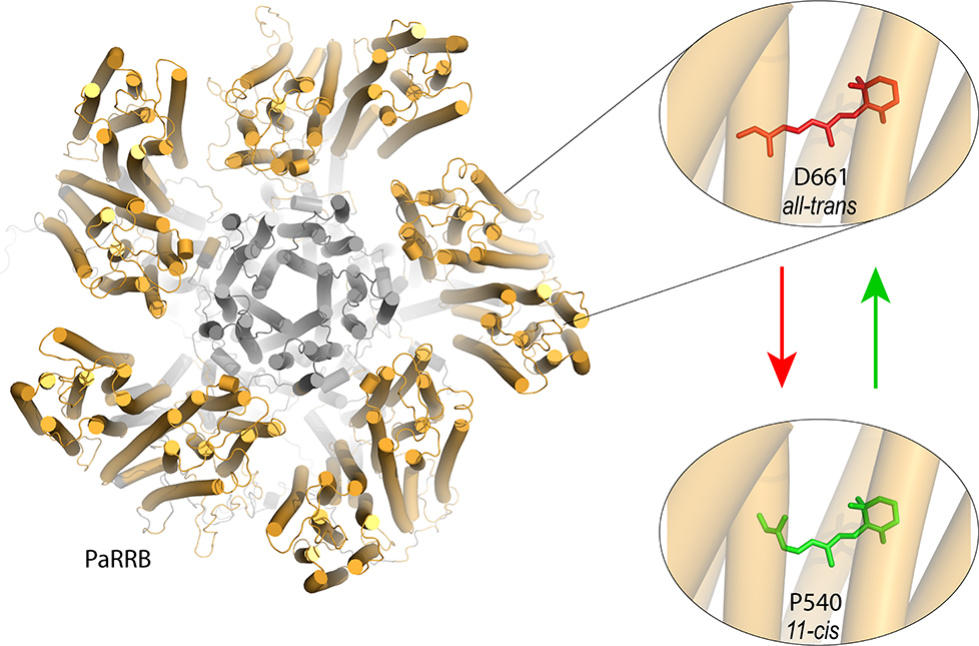
Homology model of bestrhodopsin (PaRRB) based on the structure of Tara-RRB (90% sequence identity to PaRRB)^36^ and the red-green bistability similar in both bestrhodopsins.

Femtosecond stimulated Raman spectroscopy (FSRS) is a powerful method to gain detailed molecular information on photosensory protein reaction dynamics through recording of transient vibrational spectra of the chromophore region.^43^ It features a high temporal resolution of <100 fs, high spectral resolution and a high signal-to-noise ratio to follow complex photoisomerization processes with multiple chromophore bonds involved. In contrast to traditional (time-resolved) Raman spectroscopy,^44,45^ it is insensitive to fluorescence, furthering its application scope. We have developed an FSRS method involving shot-to-shot Raman pump modulation to successfully suppress large and unpredictable baseline fluctuations and increase signal quality.^46–49^ Together with the dual electronically synchronized laser amplifier technique, a time range of femtoseconds to submillisecond becomes available and photoreceptor reaction dynamics can be studied in detail over a wide time range in a single experiment.^14,28,34,50–59^ Here, we report a transient absorption (TA) and FSRS study of the 11-*cis* to all-*trans* isomerization reaction of PaRR P540 state, a unique reaction for microbial rhodopsins, but reminiscent of animal rhodopsins. Thus, the interesting question arises whether this photoreaction in PaRR involves a direct 11-*cis* to all-*trans* photoisomerization similar to animal rhodopsins, or a multistep sequential isomerization as in the D661 forward reaction.^34^

## Results and Discussion

### Transient Absorption Spectroscopy

Here we present results on the rhodopsin tandem domain of bestrhodopsin (PaRR, residues 1–786). Figure 2A,B shows the absorption spectra and the (stimulated) Raman spectra of the D661 and P540 states, reproduced from.^34^ P540 thermally recovers to D661 on the time scale of half an hour and is therefore easily photoaccumulated with red background light. Photoaccumulated P540 can be photochemically converted to D661 with green light, rendering bestrhodopsin a quasi-bistable photochromic protein.^36^

**Figure 2:**
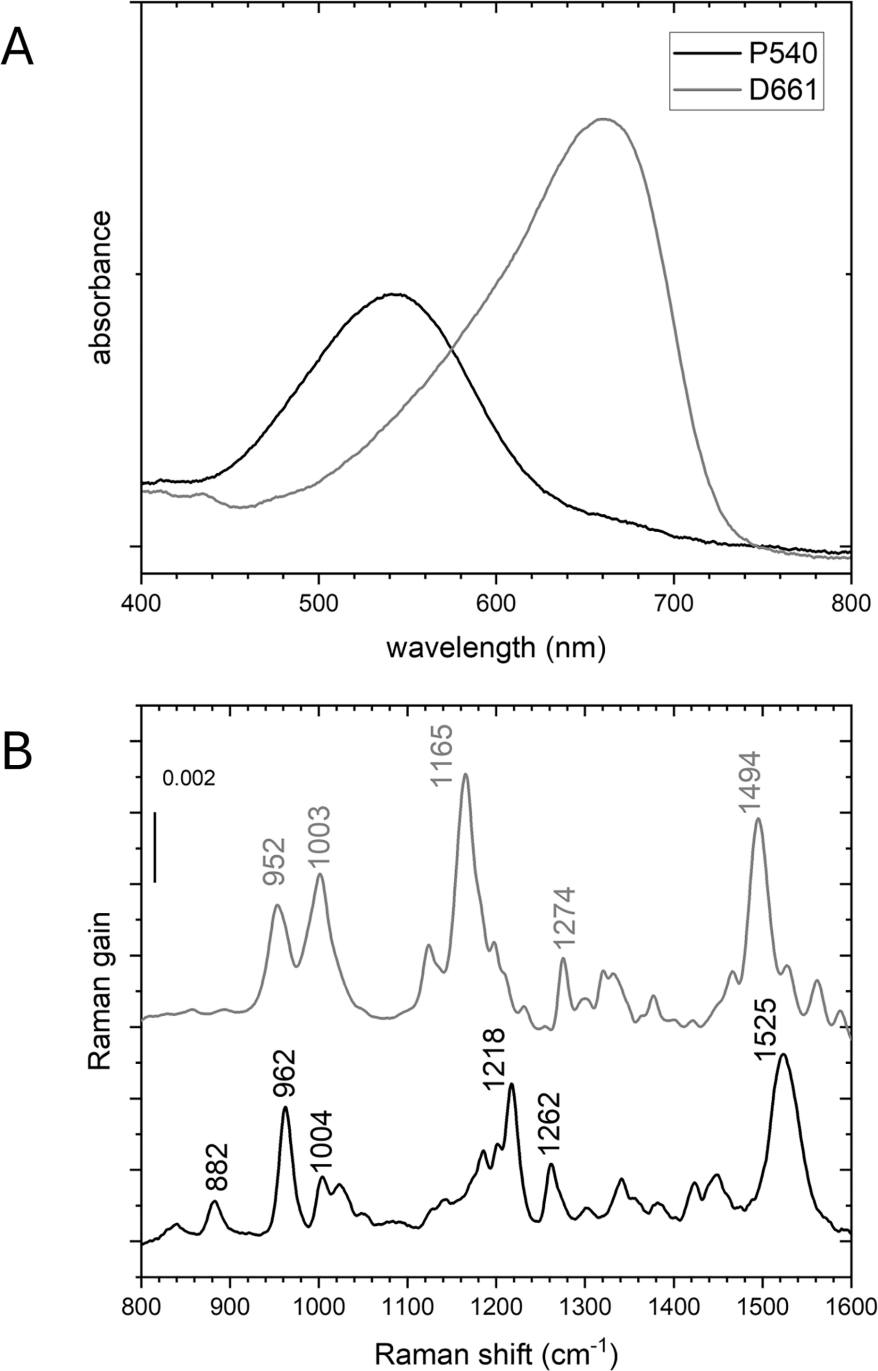
(A) Absorption spectra of bestrhodopsin D661 (gray line) and P540 (black line); (B) stimulated Raman spectra of D661 and P540, reproduced from^34^ available under a CC BY-NC-ND license. Copyright 2024 Kaziannis et al.^34^

To assess the photoreaction dynamics starting from P540, we applied ultrafast fs to subms transient absorption (TA)^60^ on photoaccumulated P540, upon excitation at 530 nm. Under continuous red background light the sample contained a 70:30 reaction mixture of P540 and D661 under 530 nm actinic excitation. The D661 contribution was subtracted from the data using the data of ref 34, yielding the pure P540 reaction dynamics. A global analysis in terms of a sequential kinetic scheme is shown in the form of evolution-associated difference spectra (EADS) in Figure S1. Figure 3A shows the results of a target analysis where the initially excited state ES1 (dark gray line) has a lifetime of 240 fs, and forms a photoproduct Q1 (red line) at a yield of ≈40%. The remainder of ES1 states evolve to a secondary excited state ES2 (medium gray line), which partly decays to the ground state (GS) and partly evolves to ES3 (light gray line) in 2.6 ps. ES3 then decays to the ground state in 45 ps. This model is similar to what we recently applied to Chrimson channelrhodopsin.^35^ The ES1-ES3 species-associated difference spectra (SADS) (Figure 3B) clearly indicate excited-state character with ground-state bleach (GSB) at 560 nm, partly compensated by excited-state absorption (ESA) at 490 nm and with stimulated emission (SE) in the region 650–775 nm. The photoproduct Q1 has a GSB at 500 nm and a product absorption band at 590 nm. Q1 evolves to the next intermediate Q2 (blue line) in 0.7 μs, with a slight but distinct spectral evolution, i.e., a blue-shift by 4 nm and a slight increase of the extinction coefficient. Q2 then evolves to Q3 (green line) in 62 μs. The quantum yield of ≈40% was estimated by reconstructing the absolute spectra of Q1–Q3 in the fs–subms TA data using the P540 absorption spectrum, as shown in Figure S3A in a procedure similar to that of the D661 state^34^ and ref 35. We observe that the target analysis with these parameters returns reasonable spectral shapes of the Q1–Q3 intermediates.

**Figure 3:**
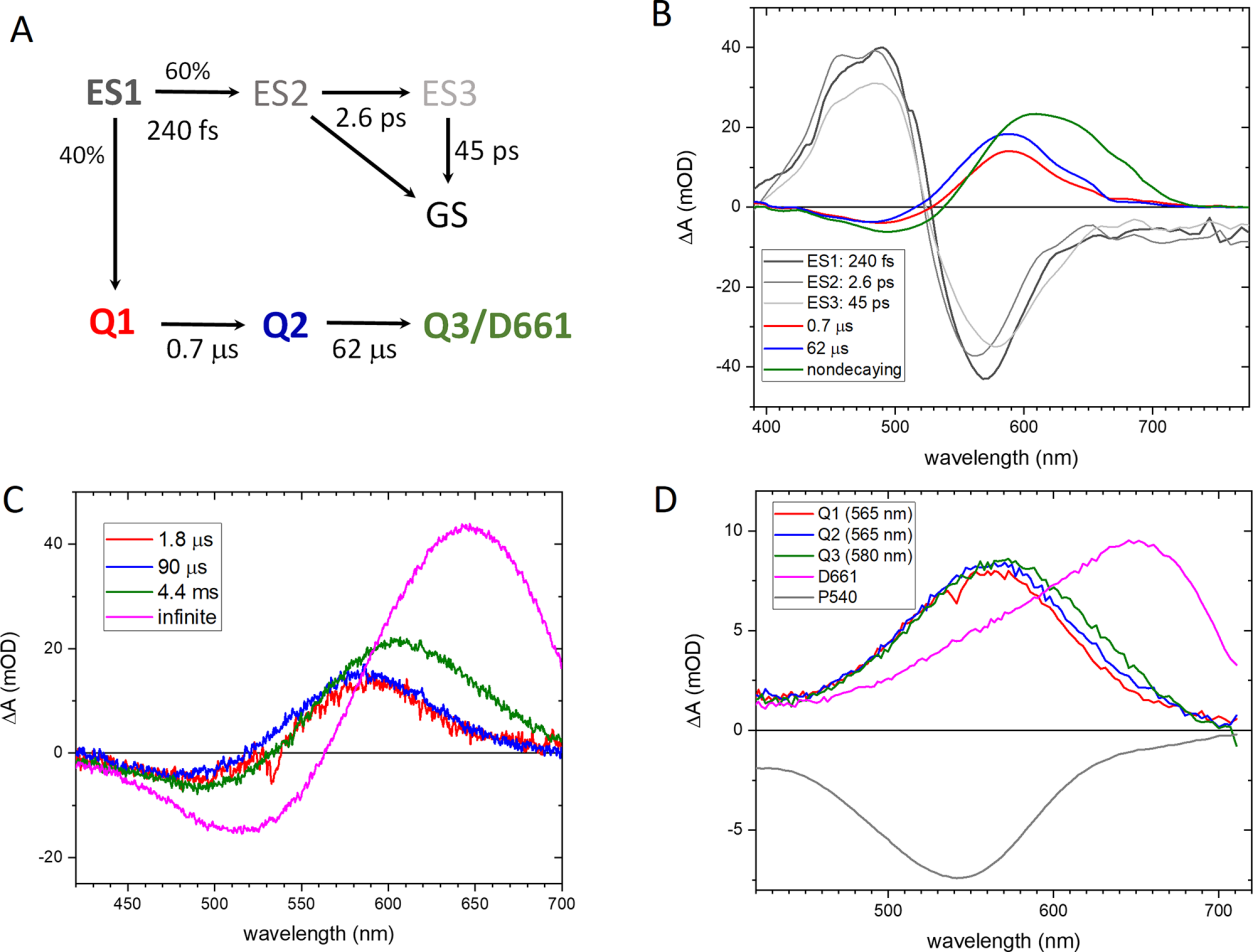
Dynamics of the photoaccumulated bestrhodopsin P540 state upon excitation at 530 nm. A: Kinetic scheme for the target analysis of the fs to subms TA data. B: SADS that result from the target analysis of the TA data with lifetimes indicated. C: EADS that follow from a sequential analysis of flash photolysis experiments of the photoaccumulated P540 state upon excitation at 535 nm. D: SAS that result from a target analysis of the flash photolysis data with the scheme indicated in Figure S3C.

The model that we applied to describe the excited-state evolution could be interpreted as branching of the initially formed excited state on the excited state PES, with one fraction (nearly) barrierlessly undergoing very fast isomerization to Q1 and the other fraction entering a nonproductive local minimum on the excited state PES, where through diffusive motions the excited state returns to the ground state on the ps to tens of ps time scale. It is unclear whether the branching occurs from the Franck–Condon region, or that it is caused by heterogeneities in the ground state. In C1C2 channelrhodopsin, such slow excited-state decay phases were assigned to nonproductive counterclockwise rotation of the reactive C13=C14 double bond with lifetimes of 2 and 11 ps, in addition to the productive clockwise rotation that lead to isomerization in 450 fs, with the multiphasic excited-state decay caused by heterogeneity in hydrogen bond network around the Schiff base.^28,61^

Flash photolysis experiments on the P540 state were consistent with the TA results (Figure 3C), with similar spectra and lifetimes of 1.8 and 90 μs. In addition, this experiment resolved the final step to the dark state D661 (Figure 3C, magenta line) in 4.4 ms. Reconstruction of the absolute absorption spectra of the photointermediates of the flash photolysis data using a sequential scheme is reported in Figure S3D. The resulting species-associated spectra (SAS) of Q1–Q3 look similar to those determined from the TA experiments (Figure S3A). We noted that in both cases, the Q3 intermediate (Figure S3A, B green lines) shows a shoulder near 650 nm, which suggests that Q3 contains a fraction of the final D661 state. This prompted us to perform a target analysis where from Q2, 75% of the population forms Q3 and 25% proceeds directly to D661 in 90 μs, as shown in Figure S3C. Indeed, clean SAS result from this approach, with maxima of Q1, Q2 and Q3 at 565, 565, and 580 nm, respectively. They are reproduced in Figure 3D.

### Femtosecond Stimulated Raman Spectroscopy

The Raman spectrum of the RSB is very sensitive to the absorption maximum, the isomeric state and the degree of distortion of the polyene backbone. The vibrational spectra of retinal have been mapped out for bacteriorhodopsin in great detail and with high confidence through a combination of resonant Raman spectroscopy, site-specific isotope labeling and quantum chemical calculations,^62–65^ and were proven to be applicable to essentially every microbial rhodopsin studied with vibrational spectroscopy to date,^14,28,66–68^ including the D661 state of bestrhodopsin.^34^ In particular, the C=C stretch frequency reports on the UV–visible absorption maximum,^67^ while the C–C stretch fingerprint region is very sensitive to the RSB isomeric state. Here, all-*trans* RSB in microbial rhodopsins is characterized by distinct bands at 1170 and 1200 cm^–1^,^65^ or at 1165 cm^–1^ in case of counterion protonation as in the bacteriorhodopsin O intermediate^62^ or the bestrhodopsin D661 state.^34^ In contrast, 13-*cis* RSB is characterized by a distinct band at 1185 cm^–1^.^63^ The unusual 11-*cis* RSB isomer in bestrhodopsin exhibits a distinct band at 1218 cm^–1^,^34,36^ reproduced for bestrhodopsin in Figure 2.^34^ Finally, the hydrogen-out-of-plane modes between 800–1000 cm^–1^ report on structural deformation of the polyene backbone at particular carbon or nitrogen atoms.^63,65^

To assess the structural dynamics of the primary and ensuing secondary products of photoactivated P540, we applied fs to subms FSRS^14,34,50^ with actinic excitation at 530 nm. Under continuous background light the PaRR sample contained a 85:15 reaction mixture of P540 and D661. The latter contribution was subtracted from the FSRS data using the data of ref 34 yielding the pure P540 reaction dynamics. The FSRS data were analyzed using the kinetic model shown in Figure 4A. Strikingly, as compared to the TA results an additional intermediate with a lifetime of 120 ps was required for an adequate fit, in addition to the 0.7 μs, 62 μs and the nondecaying components, which correspond to Q1, Q2 and Q3/D661, respectively. We denote the 120 ps component as Q0. Figure 4B shows the FSRS SADS of Q0 (black line), Q1 (red line), Q2 (blue line) and Q3/D661 (green line). Figure 4C shows a zoom-in on the C–C fingerprint region. The magenta spectrum in Figure 4B,C shows the steady-state D661 minus-P540 difference spectrum. Here, negative signals correspond to the P540 reactant state, while positive signals correspond to the product bands. The excited-state SADS of ES1, ES2, and ES3 are shown in Figure S4A and are not further considered. Figure S4B shows the excited-state and product state SADS of the D661 state reproduced from ref 34 that were used for subtraction to produce the pure P540 dynamics. Figure S5 shows a comparison between the data and fit result. Figure S6 shows the EADS of an additional data set. Figure S7 shows the results on the bestrhodopsin mutant where the conserved lysine that binds the RSB in the R1 domain was replaced by an alanine (K332A),^34^ and hence only the R2 domain binds a RSB. The FSRS EADS are nearly identical with the wild type (Figure 4B,C), which implies that the complex evolution described below does not follow from differences between the two RSBs in the bestrhodopsin tandem domain.

**Figure 4:**
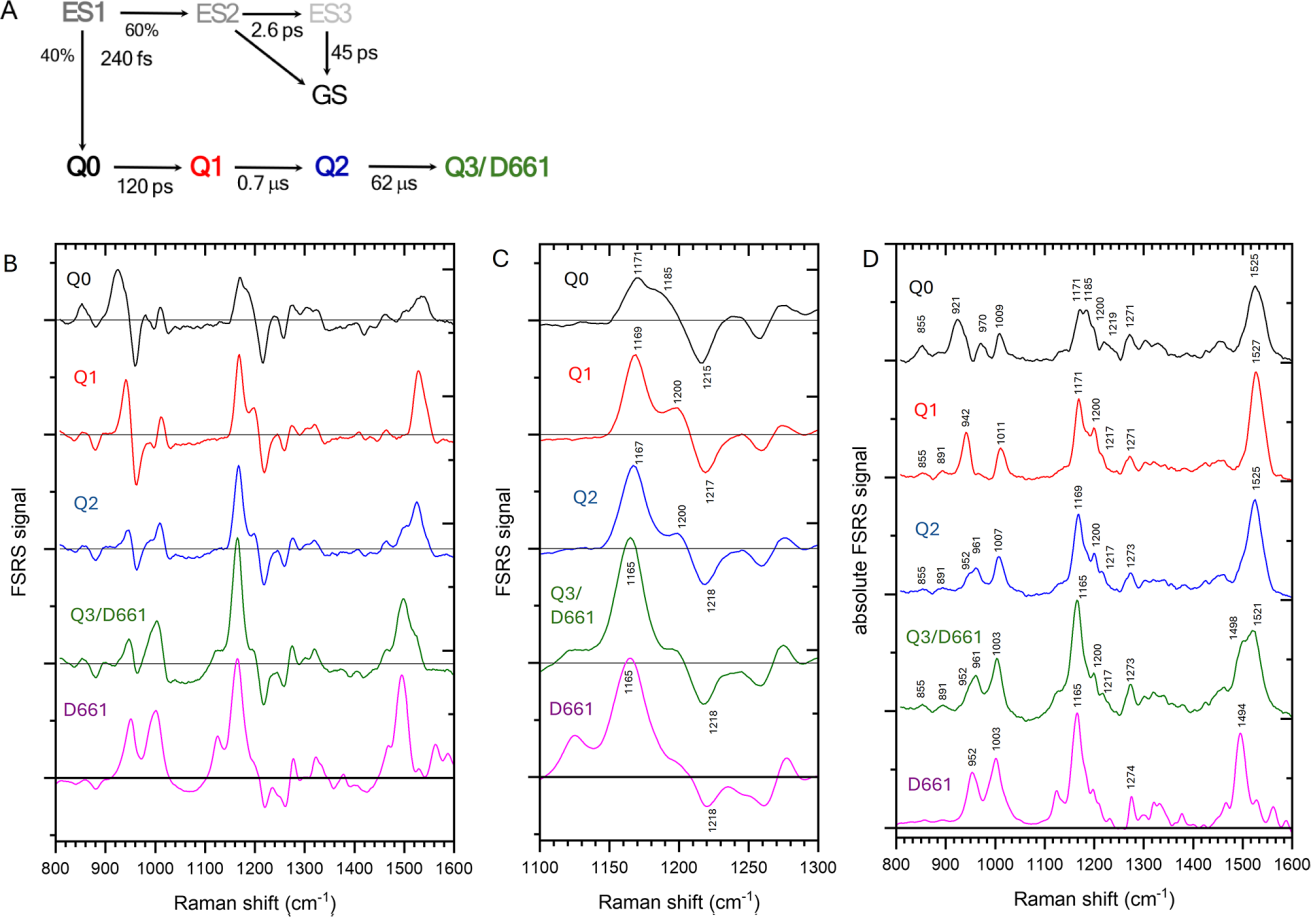
FSRS of the photoaccumulated bestrhodopsin P540 state. (A) kinetic scheme in the target analysis (B) SADS that follow from the target analysis with Q0 (black line), Q1 (red line), Q2 (blue line), Q3/D661 (green line), steady-state D661 – minus – P540 (magenta); (C) same as (B) zoomed in on the C–C stretch fingerprint region. (D) reconstructed absolute FSRS spectra of Q0 (black line), Q1 (red line), Q2 (blue line), Q3/D661 (green line), steady-state D661 (magenta).

### FSRS: RSB Isomerization Dynamics

Q0 (Figure 4B,C black line) is the primary photoproduct formed from the excited state in 240 fs. Q0 shows negative bands that closely follow the peaks of the P540 Raman spectrum of Figure 2, which is to be expected with P540 as the reactant, including a C–C stretch negative band at 1218 cm^–1^ indicative of an *11-*cis** RSB.^34,36^ Figure 4C shows a zoom in on the fingerprint C–C stretch region. Q0 features a positive band at 1171 cm^–1^, indicative of all-*trans* RSB.^65^ Strikingly, it also features a pronounced shoulder at 1185 cm^–1^, which is indicative of 13-*cis* RSB.^34,63^ To solidify the assignments of the transient FSRS spectra and facilitate comparison with the literature, we reconstructed the absolute Raman spectra of Q0 (Figure 4D, black line) and the other intermediates (Figure 4D, red, blue and green lines) by adding an appropriate amplitude of the steady-state P540 Raman spectrum (Figure 2B and ref 34 ) which was kept constant for all intermediates. Q0 shows a 1171 cm^–1^ and a 1200 cm^–1^ band pair which is typical for all-*trans* RSB in the light-adapted dark state of bacteriorhodopsin^65^ and the dark state of essentially all microbial rhodopsins characterized to date,^14,28,66–71^ indicating that 11-*cis* to all-*trans* isomerization has taken place in 240 fs. Importantly, it also shows a band at 1185 cm^–1^ which is an unambiguous marker for 13-*cis* RSB in microbial rhodopsins.^11,13,14,63,66,68,69,72,73^ Hence, the primary photoproduct Q0, formed from the excited state in 240 fs represents an isomeric mixture of all-*trans* and 13-*cis* RSB diastereoisomers. Further inspection of the Q0 Raman spectrum shows a C=C ethylenic stretch at 1525 cm^–1^, consistent with an absorption maximum at 560 nm.^67^

Q0 evolves to Q1 in 120 ps. In the Q1 SADS (Figure 4B,C, red line), the shoulder at 1185 cm^–1^ has disappeared an instead a band at 1200 cm^–1^ has come up. The absolute Q1 Raman spectrum (Figure 4D, red line) shows a prominent positive band at 1171 cm^–1^ along with a smaller band at 1200 cm^–1^ and a shoulder at 1217 cm^–1^, indicative of *all-*trans** RSB.^65^ As compared to Q0, the 1185 cm^–1^ band has disappeared, and we conclude that Q1 consists entirely of all-*trans* RSB. The amplitude ratio between the 1171 and 1200 cm^–1^ bands of Q1 (1:0.6) appears to be inverted with respect to that of all-*trans* retinal in bacteriorhodopsin and other microbial rhodopsins,^65,67^ but it should be noted that the current Raman data were taken with the FSRS technique with preresonant excitation at 800 nm, rather than traditional resonant Raman spectroscopy, which may result in variability in relative amplitudes of Raman bands. Indeed, steady-state FSRS spectra of light-adapted bacteriorhodopsin with preresonant 800 Raman excitation showed a higher 1171 cm^–1^ band as compared to the 1200 cm^–1^ band,^74^ consistent with the assignment of Q1 to all-*trans* retinal.

Inspection of the FSRS difference spectra (Figure 4B,C) reveals that the GSB signal at 1218 cm^–1^ remains constant during the Q0 to Q1 evolution (compare black and red lines), so we can exclude that the Q0 13-*cis* fraction returns to the original 11-*cis* reactant state. Instead, we conclude that the 13-*cis* RSB diastereoisomer fraction of Q0 converts to all-*trans* RSB in Q1 in 120 ps, as schematically indicated in Figure 5. The C=C ethylenic stretch mode is located at 1527 cm^–1^, essentially the same as that of Q0 (1525 cm^–1^), which implies that the Q1 absorption maximum is expected to be the similar to that of Q0, consistent with the TA results that failed to distinguish between Q0 from Q1 (Figure 3A).

**Figure 5:**
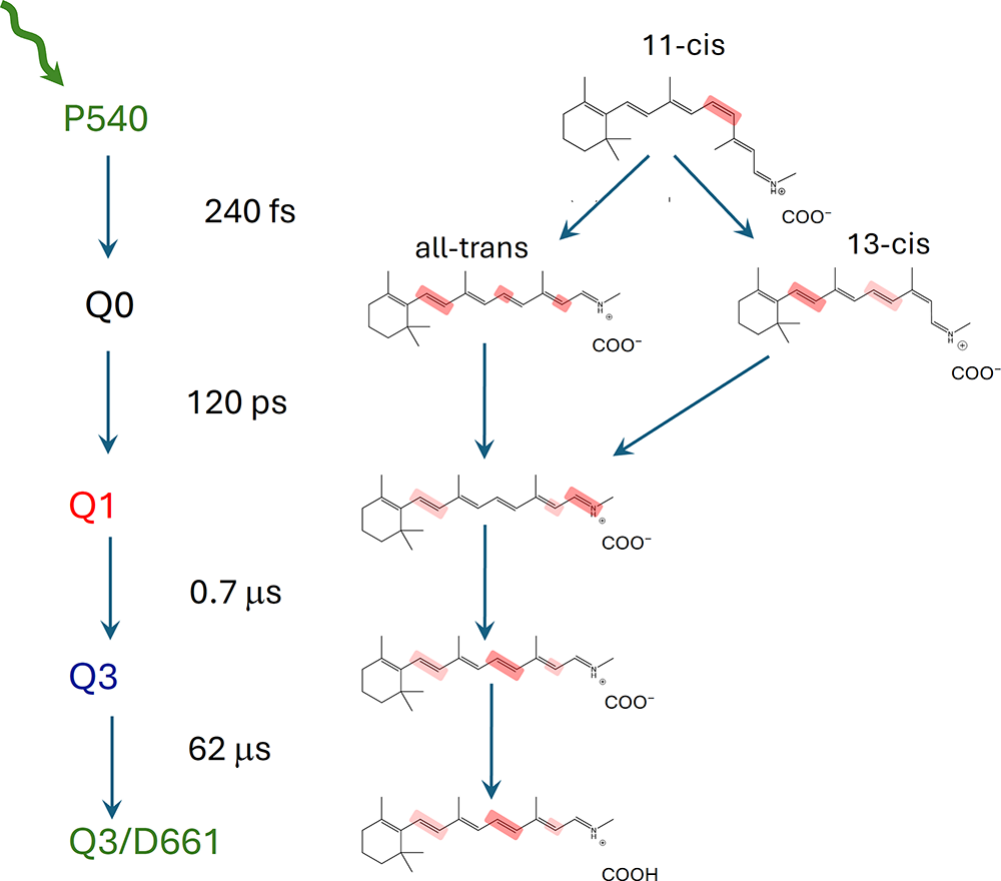
Photoreaction dynamics scheme of the bestrhodopsin P540 state with photointermediates, RSB isomeric states, RSB distortions and counterion protonation state indicated. Dark highlighting indicates strong distortion, light highlighting indicates weaker distortion.

Q1 evolves to Q2 in 0.7 μs (red to blue evolution in Figure 4B–D). The Q2 Raman spectrum is very similar to that of Q1, with almost no changes in the C–C fingerprint and C=C ethylenic stretch regions, but with significant changes in the HOOP modes, indicating distinct changes in polyene backbone distortion (elaborated on below). Q2 subsequently evolves to the Q3/D661 mixture in 62 μs (Figure 4B–D, blue to green evolution). The Q3/D661 mixture shows a double C=C ethylenic stretch band structure, with downshifted maxima at 1521 cm^–1^ and 1498 cm^–1^, consistent with a mixed population of Q3 and D661, and commensurate with the red-shift of the photointermediates as the reaction proceeds. A subtle but striking evolution on going from Q2 to Q3/D661 is observed in the C–C fingerprint region: the main band at 1169 cm^–1^ shifts down to 1165 cm^–1^, and the minor band at 1200 cm^–1^ decreases its relative amplitude in the Q3/D661 mixture state. This difference corresponds to that between the light adapted ground state of bacteriorhodopsin (BR568) and its O640 intermediate.^62,65^ The essential difference between these states is the protonation state of the counterion, with the former (BR568) deprotonated and the latter (O640) protonated. Hence, our results suggest that upon the evolution of Q2 to Q3/D661 in 62 μs, the counterion, which in fact likely forms a complex structure around the RSB in bestrhodopsin,^36^ becomes protonated. Possibly, this only applies to the D661 fraction of the Q3/D661 mixture.

### FSRS: RSB Polyene Backbone Distortion Dynamics

The FSRS spectra of Figure 4 show highly specific changes in the HOOP patterns and intensities in the Q0, Q1, Q2 and Q3/D661 intermediates, indicative of significant structural distortion dynamics of the RSB polyene backbone. Generally, HOOP modes only become symmetry allowed upon a departure of planarity of the polyene backbone,^75^ and hence provide a qualitative measure for retinal distortion, where a large HOOP amplitude indicates a high distortion, and low HOOP amplitude a low distortion. Given the extensive literature on HOOP modes in microbial and animal rhodopsins, the HOOP patterns in Figure 4 may be assigned with a moderately high degree of confidence.^63,65,75,76^ The location of the polyene backbone distortions of the Q0, Q1, Q2 and Q3/D661 as discussed in the paragraphs below are indicated in the RSB structures in Figure 5, with strong distortions indicated in dark shading, and small distortions in light shading.

In the Q0 intermediate (Figure 4D, black line), a major hydrogen-out-of-plane (HOOP) mode at 920 cm^–1^ is observed, in addition to weaker ones at 970 cm^–1^, 885 cm^–1^ and 855 cm^–1^. Assigning these modes is complex given the mixed diastereoisomer structure of Q0. However, in the bathorhodopsin intermediate of bovine rhodopsin, which binds a distorted all-*trans* RSB, a 920 cm^–1^ mode was assigned to an isolated C11H wag mode.^75^ Given that no other HOOP mode at this frequency has been reported for any RSB isomer, we therefore assign this mode to high distortion at the C11 atom in the all-*trans* diastereoisomer fraction of Q0. The 970 cm^–1^ mode may be assigned to the HC11=C12H HOOP mode in 13-*cis* RSB,^63^ indicating distortion about this double bond. The 855 cm^–1^ mode may be assigned to the C7=C8 HOOP in all-*trans* as well as 13-*cis* RSB,^63,65^ indicating distortion around this double bond, implying that the β-ionone ring assumes a twisted conformation with respect to the polyene chain. Alternatively, the 855 cm^–1^ mode may be associated with the C14H wag in all-trans RSB as in bathorhodopsin.^77^

The Q1 intermediate (Figure 4D, red line) shows a prominent HOOP band at 942 cm^–1^, which may be assigned to a NH wag—Lysine mode at the Schiff base nitrogen,^65^ indicating strong RSB distortion at the Schiff base. A similar band was observed for the early strongly distorted intermediates of the bestrhodopsin D661 photoreaction.^34^ The 855 cm^–1^ HOOP has diminished in amplitude with respect to Q0, indicating a relaxed distortion around the C7=C8 double bond or at the C14 atom.

In the Q2 intermediate (Figure 4D, blue line), the HOOP band at 942 cm^–1^ in Q1 has shifted to 961 cm^–1^ along with a shoulder at 952 cm^–1^, where the former can be assigned to the C11H=C12H HOOP mode,^65^ which indicates that the structural distortion of the RSB moves from the Schiff base region to the C11=C12 bond. In the Q3/D661 intermediate, the HOOP modes remain nearly the same.

### The Mechanism of the Multistep 11-*cis* to All-*trans* Isomerization

Figure 5 shows an overview of the bestrhodopsin P540 photoreactions, which involve a highly unusual multistep isomerization process from the 11-*cis* reactant, with a fraction of directly formed all-*trans* RSB and a fraction of directly formed 13-*cis* RSB, together forming the primary photoproduct Q0. The 13-*cis* diastereoisomer fraction of Q0 then thermally isomerizes to all-*trans* in 120 ps, while concomitantly both the all-*trans* and 13-*cis* diastereoisomer fractions of Q0 undergo structural changes of the polyene backbone forming the Q1 intermediate. These multiple processes do not necessarily occur with identical time constants, but rather with time constants that are sufficiently close to be lumped in a single time constant in the global analysis procedure.

To rationalize the formation of both all-*trans* and 13-*cis* photoproducts, we first note that in microbial rhodopsins and animal rhodopsins alike, isomerization about double bonds proceeds through a volume-conserving aborted bicycle pedal mechanism,^19,21,22,78,79^ whereby photoexcitation results in corotation in opposite directions of the C9=C10, C11=C12 and C13=C14 double bonds in varying degrees, depending on the rhodopsin type. The aborted rotation of either of the double bonds then leads to a net unitary double-bond isomerization to only a single diastereoisomer, i.e., only about C11=C12 in animal rhodopsins and only about C13=C14 in most microbial rhodopsins.

We propose that in the bestrhodopsin P540 state, photoexcitation of the 11-*cis* chromophore results in corotation of the C11=C12 and C13=C14 double bonds (Figure 6A), whereby the excited state population splits into two fractions together constituting the Q0 intermediate: fraction 1 performs an aborted bicycle pedal motion passing through the C11=C12 conical intersection with abortion of the C13=C14 rotation, resulting in a distorted all-*trans* structure (Figure 6B, green trajectory). Fraction 2 performs a full, nonaborted bicycle pedal motion, passing through the C13=C14 conical intersection while not aborting the C11=C12 rotation, resulting in a distorted 11-*trans*, 13-*cis* chromophore (Figure 6, red trajectory). These processes occur (near)-barrierless given the fast isomerization rate constant of (240 fs)^−1^. In both the all-*trans* and 13-*cis* diastereoisomer fractions, steric interactions with amino acid side chains of the binding pocket cause the chromphores to become trapped close to the conical intersection in shallow potential energy wells, resulting in a restricted conformational space and structurally similar, highly distorted chromophore structures separated by low energetic barriers. Consistent with this notion, the HOOP modes in the Q0 intermediate show a high amplitude (Figure 4D, black line), indicative of an extensively distorted polyene backbone. This model would imply similar chromophore structures for the respective formal all-*trans* (green PES well) and 13-*cis* diastereoisomers (red PES well) and allow thermal conversions from the 13-*cis* to all-*trans* isomers given the low energetic barriers that would consequently exist. The conical intersection region likely forms a seam connecting the excited-state and ground-state PES, rather than the distinct conical intersections as drawn in Figure 6, which would imply that the C13=C14 and C11=C12 conical intersections cannot be distinguished.^80^

**Figure 6:**
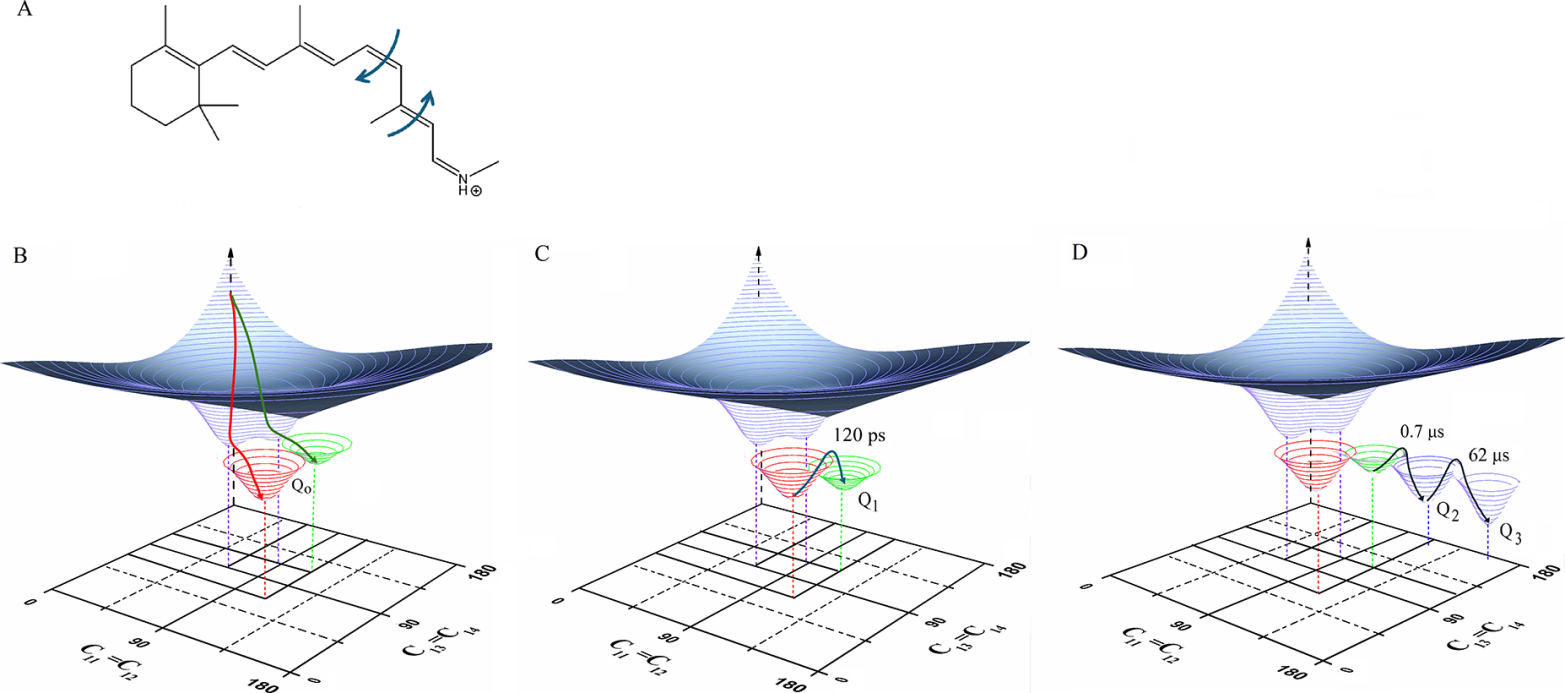
(A) 11-*cis* RSB and rotations about the C11=C12 and C13=C14 double bonds in the bicycle pedal isomerization mechanism. (B) Schematic potential energy surface (PES) for bestrhodopsin P540, branching of excited states into the conical intersection to all-*trans* via an aborted bicycle pedal mechanism (green pathway) and to 13-*cis* via a full bicycle pedal mechanism (red pathway) to form Q0, which consists of a mixture of all-*trans* and 13-*cis* diastereoisomers. After passing through either CI, the chromophore becomes structurally trapped close to the conical intersections high up on the ground state PES. (C) same as (B), with the 13-*cis* chromophore fraction of Q0 converting to all-*trans* on the ground state PES in 120 ps to form Q1 (dark blue pathway). (D) same as (B) and (C), with Q1 structurally relaxing along the C11=C12 and C13=C14 reaction coordinates toward Q2 and Q3 on the 0.7 and 62 μs time scale. The C11=C12 and C13=C14 angles are indicative and by no means quantitative.

The evolution of Q0 to Q1 in 120 ps represents thermal isomerization of the 13-*cis* diastereoisomer fraction of Q0 to all-*trans* RSB, as indicated in Figure 6C. In addition, the strain in the C7=C8 double bond is released, implying the β-ionone ring tilt has relaxed. The strain at the C11 atom of the all-*trans* RSB fraction and the strain at the C13=C14 double bond is released and transferred to the C=N-Lys bonds, which becomes highly distorted. We propose that as a result, the potential energy well of all-*trans* RSB (Figure 6C, green well) lowers in energy allowing the 13-*cis* diastereoisomer fraction of Q0 to thermally convert to all-*trans* in 120 ps (Figure 6C, black arrow).

The evolution of Q1 to Q2 in 0.7 μs and to Q3/D661 in 62 μs represents further conformational and energetic relaxation of the all-*trans* RSB enabled by protein relaxation, consistent with the overall lower amplitude of the HOOP modes, as indicated by the blue arrows in Figure 6D.

The model presented above provides for an understanding why Q0 and Q1 have identical absorption spectra, which is an unusual result given that Q0 contains all-*trans* and 13-*cis* retinal, while Q1 contains only all-*trans*. In many cases, different diastereoisomers exhibit distinct absorption spectra,^1,63,65^ although in channelrhodopsin-2 (CrChR2) and HKR1 rhodopsin, all-*trans* and 13-*cis* diastereoisomers absorb very similarly.^50,70,81^ UV–vis absorption spectra for distinct diastereoisomers are usually measured in steady-state for structurally relaxed diastereoisomers, which are widely separated on the ground state potential energy surface and hence probe entirely different regions of the excited state PES through their Franck–Condon regions. Here, the Q0 and Q1 all-*trans* and 13-*cis* diastereoisomers are unrelaxed and structurally similar, located closely together on the ground state potential energy surface (Figure 6B, left and middle panel), hence probing similar parts of the excited-state PES. This implies that their absorption spectra may well be indistinguishable. In fact, the retinal C=C stretch frequencies of Q0 and Q1 are nearly identical at 1525–1527 cm^–1^ (Figure 4D) which implies that they may have identical absorption maxima: the C=C stretch is a measure of electron delocalization over the polyene backbone, which determines the absorption spectrum.

Our results on the multistep 11-*cis* to all-*trans* isomerization in bestrhodopsin P540 are reminiscent of our previous results on the multiple isomerizations of the bestrhodopsin D661 state,^34^ where a mixture of 11-*cis* and 13-*cis* diastereoisomers as characterized by the same fingerprint bands as observed in this study at 1218 and 1185 cm^–1^, respectively, was formed from the all-*trans* dark state on the picosecond time scale. Subsequent thermal isomerization of the 11-*cis* diastereoisomer fraction to 13-*cis* occurred in 500 ps, followed by the reverse 13-*cis* to 11-*cis* thermal isomerization process on the μs time scale. As we did here, this phenomenon was rationalized by the notion that the early photoproducts would exhibit corotation of their C11=C12 and C13=C14 double bonds according to the aborted bicycle pedal model^78^ thereby proceeding through distinct conical intersections to produce both 11-*cis* and 13-*cis* RSB, and become trapped high up the potential energy surface close to the conical intersection. This model would imply similar RSB structures of the respective formal 11-*cis* and 13-*cis* conformers and allow thermal conversions between the 11-*cis* and 13-*cis* diastereoisomers given the low energetic barriers that would consequently exist.^34^ Dynamic steric conflicts of RSB in the protein binding pocket would force the RSB in highly distorted intermediates, and only upon protein relaxation the RSB could structurally and energetically relax to a single stable 11-*cis* photoproduct.^34^ Support for the possible occurrence of such processes was provided by recent computational studies on heliorhodopsin, which predicted extensive C11=C12 and C13=C14 corotation, even if the C11=C12 rotation was finally aborted.^82^

One might consider the possibility that the 1185 cm^–1^ band does not relate to the transient occurrence 13-*cis* RSB, but rather to all-*trans* RSB that has its frequency shifted due to the unusual binding pocket of bestrhodopsin which favors 11-*cis* rather than 13-*cis* RSB. However, this proposition is internally inconsistent because the 1185 cm^–1^ band occurs as a transient intermediate in both the D661 ^34^ as the P540 photoreactions (this work). Given that all-*trans* and 11-*cis* RSB represent the reactant in the former and latter case, respectively, the 1185 cm^–1^ band cannot be associated with either of them, leaving 13-*cis* RSB as the only reasonable assignment, in line with the literature.^13,63,66–69^

### Comparison of 11-*cis* to All-*trans* Isomerization Dynamics of Bestrhodopsin with Bovine Rhodopsin

The bestrhodopsin P540 state constitutes the only microbial rhodopsin of which the photochemistry involves a 11-*cis* to all-*trans* RSB isomerization, and it is hence of interest to compare the results with those of animal rhodopsins, in particular bovine rhodopsin which exhibit such an isomerization as well.^3,12,21,79,83^ It should be noted that the β-ionone rings are oriented differently about the C6–C7 single bond in microbial rhodopsin and bovine rhodopsin,^84–86^ so the RSB diastereoisomer structures of bestrhodopsin P540 and bovine rhodopsin are not identical. Even if the reactant and final product states are similar, fundamental differences exist between the two photoreactions. Bovine rhodopsin exhibits an ultrafast 11-*cis* to all-*trans* isomerization reaction that is exceedingly fast and ballistic^3,5^ caused by direct curve crossing that is complete in less than 200 fs at an efficiency of 65%.^2,4,21,25,26,87^ The amino acids in the binding pocket shield the electrostatic effects of the counterion during the isomerization process,^87,88^ allowing the reaction to proceed at exceptional speed and efficiency. In contrast, the bestrhodopsin P540 state shows an ultrafast branched isomerization in 240 fs, forming a direct all-*trans* RSB fraction and a 13-*cis* RSB fraction at a total yield of about 40%. Even if this reaction may be (nearly) barrierless given the fast reaction rate, the reaction coordinate must be significantly different from that of bovine rhodopsin given the different diastereoselectivities. Most likely, (dynamic) steric interactions with particular amino acid side chains determine whether the outcome of the reaction is all-*trans* or 13-*cis*, i.e., whether it enters an aborted bicycle pedal motion to the all-*trans* or a full bicycle pedal motion to the 13-*cis* diastereoisomer. The all-*trans* fraction of Q0 shares some similarities with rhodopsin’s bathorhodopsin in the sense that the same HOOP mode at 920 cm^–1^ is activated (Figure 4D), indicating strong distortion at the C11 atom. In addition, the 13-*cis* fraction is unstable and trapped close to the conical intersection to allow for thermal isomerization to all-*trans* RSB. While in bovine rhodopsin the primary photoproduct bathorhodopsin represents a highly distorted intermediate as well,^12,75,89^ it is diastereoisomerically stable at all-*trans* and evolves to Lumirhodopsin at a 100% quantum yield. The excited-state PES of bestrhodopsin P540 differs significantly from that of bovine rhodopsin given that 60% of the excited states show diffusive nonproductive motion with resulting picosecond lifetimes. In bovine rhodopsin, only a very small picosecond phase was observed in the excited-state decay.^90^

The proposed mechanism for bestrhodopsin P540 with bifurcation of the excited state population into an aborted and nonaborted bicycle pedal motion, leading to mixed diastereoisomers (Figure 6B) is reminiscent of the dynamics of isorhodopsin, which is a particular state of bovine rhodopsin which binds 9-*cis* RSB.^21,91^ In isorhodopsin, upon photoexcitation a fraction undergoes isomerization from 9-*cis* to an all-*trans* photoproduct via an aborted bicycle pedal motion involving C9=C10–C11=C12. Via the same conical intersection a small fraction undergoes a full bicycle pedal motion, resulting in 11-*cis* RSB, the native rhodopsin form. Full bicycle pedal motions were observed *ab initio* computationally in RSB model systems.^92–94^

## Conclusion and Perspective

Possibly, the mechanism proposed here for simultaneous production of two different diastereoisomers may generally apply for rhodopsins where the production of only one diastereoisomer, as in most animal and microbial rhodopsins, may be regarded as limiting cases. In fact, the initially created excited state population may be sufficiently broadly distributed to enter the intersection space in a large segment. Such segment may be wide enough to contain conical intersection points leading to different photoproducts^80^ such as we observed here for bestrhodopsin P540 and bestrhodopsin D661,^34^ and was observed for isorhodopsin.^91^ Very likely, in most rhodopsins steric constraints imposed by the RSB binding pocket restrict the bicycle pedal motions, resulting in single diastereoisomer selectivity. Consistent with this idea, a reanalysis^20^ of fs XFEL data on bacteriorhodopsin^17^ demonstrated that the all-*trans* retinal undergoes extensive isomerization sampling about multiple double bonds before it proceeded to the productive 13-*cis* diastereoisomer.

## Experimental Section

### Sample Preparation

Bestrhodopsin samples were prepared as described previously.^34^ The tandemly arranged rhodopsin sequences (residues 1–786; GenBank: USH44360.1 derived from CCMP1374. The DNA provided by Dr. M. Shalev-Benami, Weizmann Institute of Science, Israel) was cloned into a pPICZ vector (Thermo Fisher Scientific, Waltham, MA, USA) with C-terminal StrepII-Tag. cells were transformed, and recombinant clones were selected according to the instructions of the manufacturer (Thermo Fisher Scientific, Waltham, MA, USA). Protein expression in buffered growth medium supplemented with 5 μM all-*trans* retinal was induced by adding 2.5% (v/v) methanol, and cells were harvested 24 h postinduction and lysed by a high-pressure homogenizer (HTU Digi-French-Press, G. Heinemann, Germany). The recombinant rhodopsin-tandem was solubilized from crude membrane fraction with 1.5% (w/v) dodecylmaltoside (DDM, Glycon, Luckenwalde, Germany) in HBS-buffer (50 mM HEPES pH 7.4, 100 mM NaCl). The protein was purified by affinity-chromatography using a 5 mL Strep-TactinXT Superflow column (IBA GmbH, Göttingen, Germany), washed with 50 mL of washing buffer (50 mM HEPES (pH 7.4), 100 mM NaCl, 0.02% DDM), and eluted with 2× BTX-buffer containing 100 mM biotin (IBA GmbH) and supplemented with 0.02% DDM. Colored fractions were pooled and concentrated in a washing buffer (Amicon Ultra Centrifugal Filter, molecular weight cutoff (MWCO) 100 kDa, Merck Millipore, Burlington, MA, USA). The lysine mutant (K332A) was obtained by site-directed mutagenesis (using the following Primers: GACGTGGTCATGGCGCTGTCCCACACC; GGTGTGGGACAGCGCCATGACCACGTC), then further prepared and measured analog to the wildtype.^34^

### Transient Absorption Spectroscopy

All time-resolved-spectroscopy experiments^60^ were conducted on a home-built 1 kHz transient absorption (TA) and femtosecond-stimulated Raman spectroscopy (FSRS) set-ups constructed around femtosecond titanium:sapphire amplifiers Femtopower (Spectra Physics) and Solstice amplifier (Spectra Physics) sharing a common oscillator. The amplifiers were synchronized both by means of electronic triggering and optical delay of the seed prior to amplification, which allows for setting the delay between their pulses up to <1 ms with femtosecond precision. In the TA experiment two laser beams were employed, pump and probe. A white light supercontinuum generated in an argon-filled hollow core fiber (Savannah, Ultrafast Innovations, Germany) driven by the Femtopower amplifier served as the probe. The pump beam (centered at 520 nm, 50 nJ per pulse) was generated by an optical parametric amplifier (OPA) (TOPAS, Light Conversion, Vilnius, Lithuania) driven by the Solstice amplifier. Pump and probe beams were overlapped and focused at the same spot in the sample. Each beam was interrupted on shot-to-shot basis by an optomechanical chopper to acquire all four possible pulse combinations (pumped, not-pumped, dark background, pump-only). The spectrum of the probe beam transmitted through the sample was acquired by a home-built prism spectrometer utilizing 1 kHz CCD camera (Entwicklungsbuero Stresing, Berlin, Germany). In order to reduce the noise due to white light fluctuations we used a second identical detector to acquire a reference spectrum of the probe replica avoiding the sample and we performed a correction as described in ref 95. During all experiments the sample was kept in a 1 mm thick optical cell. The common focus of laser beams was randomly scanned across the sample surface in order to replenish fresh sample. Additionally, the sample was continuously illuminated with a red LED in order to facilitate the bestrhodopsin conversion to the P540 state. In total 351 time delays were acquired, the first 151 delays sampling the coherent artifact region with 20 fs steps, the rest exponentially sampling the photoinduced dynamics of Bestrhodopsin up to 0.3 ms. All the experiments were taken under the magic angle (54.7°) condition to remove the influence of orientation relaxation. A fraction of D661 remained in the sample, which was taken into account in the global analysis procedure by subtracting out the D661 dynamics published in.^34^

### Flash Photolysis Spectroscopy

Submicrosecond-to-second transient absorption spectra (flash photolysis) were obtained with LKS.60 flash photolysis system (Applied Photophysics Ltd., Leatherhead, UK) equipped with a tunable optical parametric oscillator (MagicPrism, Opotek Inc., Carlsbad, CA, USA) pumped by the third harmonic of an Nd:YAG laser (Brilliant B, Quantel, Les Ulis, France). The protein was excited with a 10 ns laser pulse, adjusted to 535 nm, and probed by the light of a short-arc XBO xenon lamp (150 W, Osram, München, Germany). The transient absorbance changes (10 ns to 1 s) were detected by an Andor iStar ICCD Camera (DH734, Andor Technology, Belfast, Northern Ireland), and the data was averaged over 19 cycles. Photoinduced conversion to P540 was achieved by illuminating the sample for ∼1 s with 660 nm LED light after each taken spectrum.

### Femtosecond Stimulated Raman Spectroscopy

The femtosecond-stimulated Raman spectroscopy setup is an upgraded version^51^ of the one described in our previous work.^46^ In the current design, we seeded two independent 1 kHz chirped pulse amplifiers (CPAs) with femtosecond pulses from one shared Ti: sapphire oscillator. We delayed the seed electronically and optically to trace processes beyond six nanoseconds. To initiate a photoreaction, we employed pulses of 50 nJ at 530 nm (data set I, shown inFigure [Fig fig4]), 200 nJ at 515 nm (data set II, shown in Figure S6) or 200 nJ (K332A mutant) from the OPA driven by the Solstice amplifier focused into a 100 μm spot as the actinic pump. Actinic pulse duration was about 50 fs (full width half-maximum). Meanwhile, we focused a 1450 nm signal beam from a second OPA system on a moving CaF_2_ plate to generate a white light supercontinuum as the probe, and the probe was focused on the sample at a spot of approximately 50 μm. In the detection apparatus, the probe was split into two beams. One part was sent to a grating-based high resolution imaging spectrograph (Acton, Princeton instruments) for Raman analysis in the 750 to 950 nm region. The other part was directed to a prism spectrograph to obtain transient absorption spectra in the 370 to 1200 nm range. In both spectrographs, a 58 × 1024 pixels CCD camera (Entwicklungsbuero Stresing) was used as a linear image sensor via operation in a full vertical binning mode. Cameras were triggered from the lasers at 1 kHz and provided shot-to-shot detection. The 800 nm femtosecond pulses from the second amplifier passed through a home-built pulse shaper to create a series of frequency-locked picosecond pulses as the Raman pump, totaling 96 wavelength-shifted Raman pumps. The energy of the Raman pump was 2 μJ for data set I presented in Figure 4, 3.8 μJ for data set II presented in Figure S6, and 9 μJ for the Lys mutant K332A presented in Figure S7. We implemented 76 exponentially spaced time delays from −1 ns to 0.4 ms with 50 fs steps around time zero to sample the photoinduced dynamics of bestrhodopsin. All the experiments were taken under the magic-angle (54.7°) condition to remove the influence of orientation relaxation. To reduce the impact of photodamage in the measurement, we moved the sample in the beam at a speed of approximately 10 cm/s in a sample scanner. The sample was contained in a cuvette is 1 mm path length and kept in the P540 state by background illumination with a red LED. A certain fraction of D661 remained in the samples, which was taken into account in the global analysis procedure with the following approach. We collected FSRS data with red background illumination, resulting in a ≈85:15% mixture of P540:D661, and FSRS data with green background illumination (recorded in the same session or published in ref 34, containing a 100% D661 sample). We then performed a simultaneous analysis of the P540 + D661 and D661-only FSRS data, thereby manually varying the fraction of D661 in the P540 + D661 data set, and determining if the resulting P540 FSRS spectra were reasonable, i.e., that they did not contain any D661 features. The low D661 fraction resulting from this analysis (≈15%) gives confidence that that the P540 FSRS spectra are reliable.

### Global and Target Analysis

The general target analysis methodology has been described in ref 96. The global analysis of the FSRS data was performed as described in ref 97. Here, the perturbed free induction decay (PFID) that characterizes the FSRS signals picoseconds before time zero has explicitly been quantitatively taken into account, which allows the extraction of very short time constants in the order of 100 fs in the genuine spectral evolution after time zero with high confidence.

## Supplementary Material



## Notes

Sequential analysis of TA data, TA kinetic traces and result of target analysis, kinetic scheme for target analysis and SAS of TA and flash photolysis data, FSRS SADS that result from simultaneous target analysis of D661 and P540 states, heat map of raw FSRS data along with fit result, FSRS EADS of second data set, FSRS SADS of Lysine mutant K332A (PDF)
