# Supplementary material for: Multistep 11-cis to All-trans Retinal Photoisomerization in Bestrhodopsin, an Unusual Microbial Rhodopsin
Source: J Am Chem Soc. 2025 Jul 10;147(29):25571–83. doi: 10.1021/jacs.5c06216 (PMC12291454; doi:10.1021/jacs.5c06216)
Supplement: Supplementary file 1 [file ja5c06216_si_001.pdf]

Supporting information

Multi-step 11-*cis* to all-*trans* retinal isomerization in bestrhodopsin, an unusal microbial rhodopsin

Matthias Broser[1¶], Spyridon Kaziannis[2,3¶], Ivo H.M. van Stokkum[4], Atripan Mukherjee,[2] Jakub Dostal[2], Wayne Busse[1], Arno Munhoven[1], Cesar Bernardo[2], Peter Hegemann[1], Miroslav Kloz,[2] John T.M. Kennis[4*]

[1]Institut für Biologie, Experimentelle Biophysik, Humboldt Universität zu Berlin, Invalidenstrasse 42, D-10115 Berlin, Germany
[2]ELI-Beamlines, Institute of Physics, Na Slovance 2, 182 21 Praha 8, Czech Republic
[3]Department of Physics, University of Ioannina, Ioannina, Gr-45110
[4]Department of Physics and Astronomy, Vrije Universiteit Amsterdam, Amsterdam 1081 HV, De Boelelaan 1081, The Netherlands

[¶]these authors contributed equally
*corresponding authors, j.t.m.kennis@vu.nl

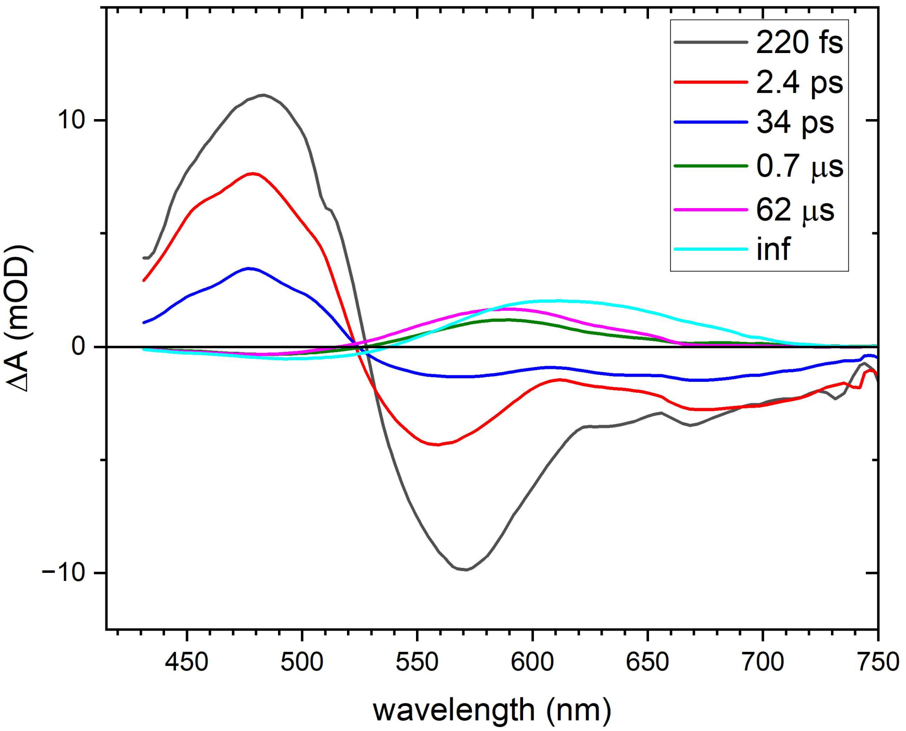

Fig. S1. EADS that follow from a sequential analysis of the TA experiment on *P. antarctica* bestrhodopsin P540, with time constants indicated. The 220 fs EADS (black line) shows ground state bleach (GSB) and stimulated emission (SE) in the 550 - 750 nm region, and excited-state absorption (ESA) at 470 nm, indicating that the 220 fs EADS corresponds to the excited state. It evolves in 220 fs to the red EADS, which shows a diminished ESA and GSB/SE, indicating that a fraction of the excited states has decayed. In addition, the spectral shape is different from that of the black EADS, with an (apparently) dimished GSB signal around 570 nm with respect to the SE signal around 650 nm, which suggests that the GSB has been compensated by a product absorption. The evolution in 2.4 ps to the blue EADS indicates a further decrease of the excited-state signals, along with a larger relative decrease of the GSB at 570 nm, giving further evidence for product absorption in that spectral region. In the next evolution in 34 ps to the green EADS product absorption around 580 nm is apparent, along with GSB around 500 nm. The product band then evolves in 0.7 μs to the magenta EADS, which in turn evolves to the non-decaying EADS (cyan line) in 63 μs.

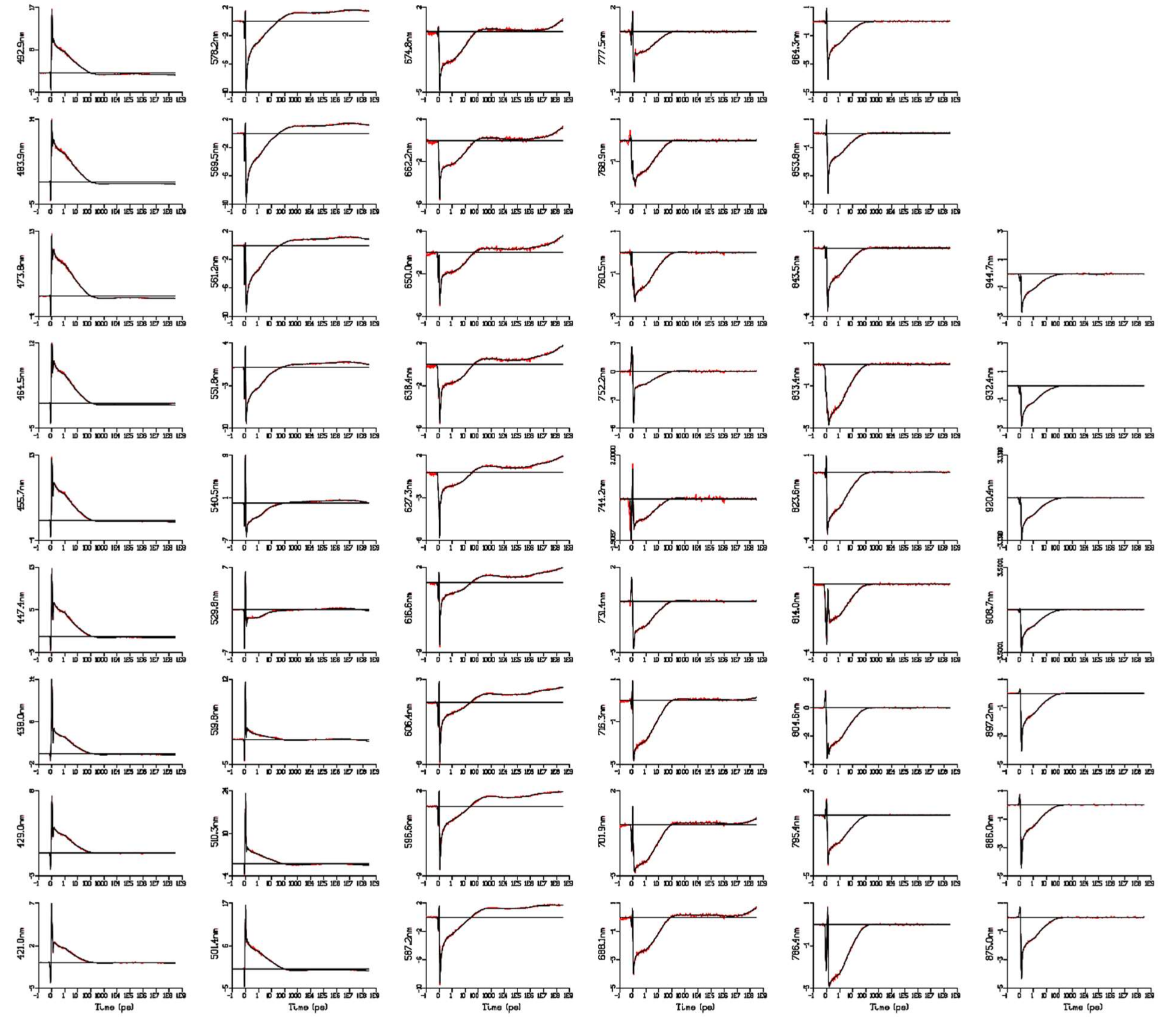

Fig S2. TA kinetic traces (in red) at wavelengths indicated in the ordinate labels along with the result of the target analysis (black). Note that the time axis is linear until 1 ps after the maximum of the IRF, and logarithmic thereafter.

A

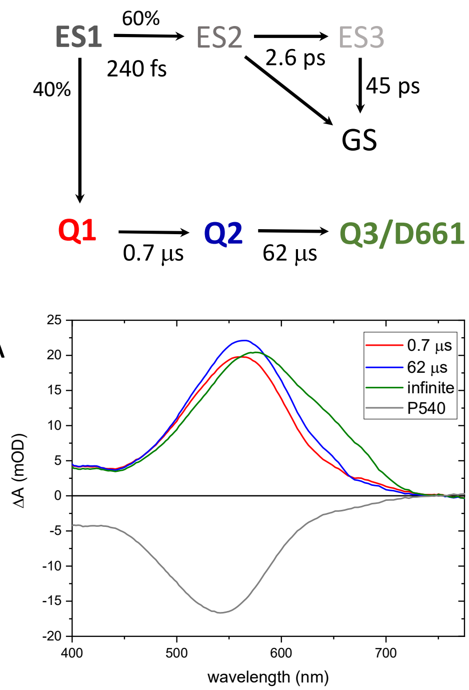

A: SAS of the TA data on the green absorbing P540 state as determined from a target analysis with the scheme indicated, with Q1, Q2 and Q3 with lifetimes indicated. The P540 absorption spectrum is shown negative.

B

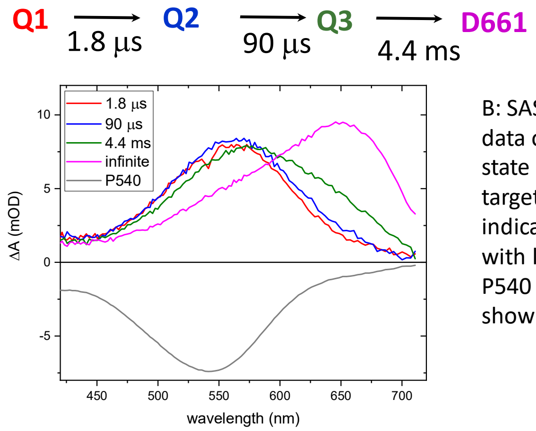

B: SAS of the flash photolysis data on the green absorbing state as determined from a target analysis with the scheme indicated, with Q1, Q2 and Q3 with lifetimes indicated. The P540 absorption spectrum is shown negative.

C

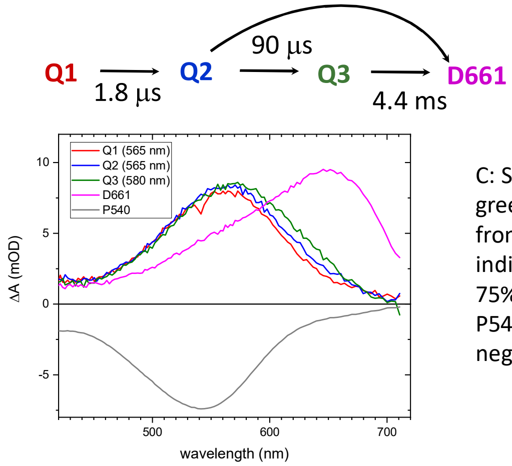

C: SAS of the flash photolysis data on the green absorbing state as determined from a target analysis with the scheme indicated, where Q2 proceeds to Q3 by 75% and to D661 by 25% in 90 μs. The P540 absorption spectrum is shown negative.

Fig. S3

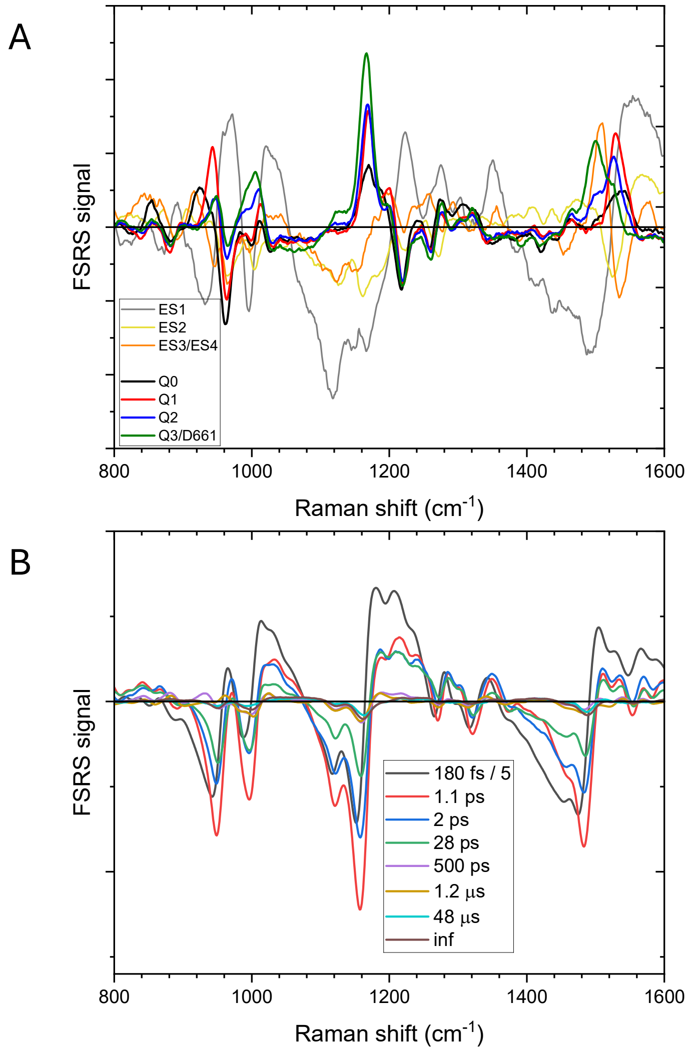

Fig. S4. A: excited and product state SADS for the *P. antarctica* bestrhodopsin P540 state using the kinetic scheme shown in Fig. 4A. Q0, Q1, Q2 and Q3/D661 were reproduced from Fig. 4B. (B) EADS from a sequential analysis of the D661 data, reproduced from ref.[1] Note that the amplitude of the 180 fs EADS was divided by a factor of 5.

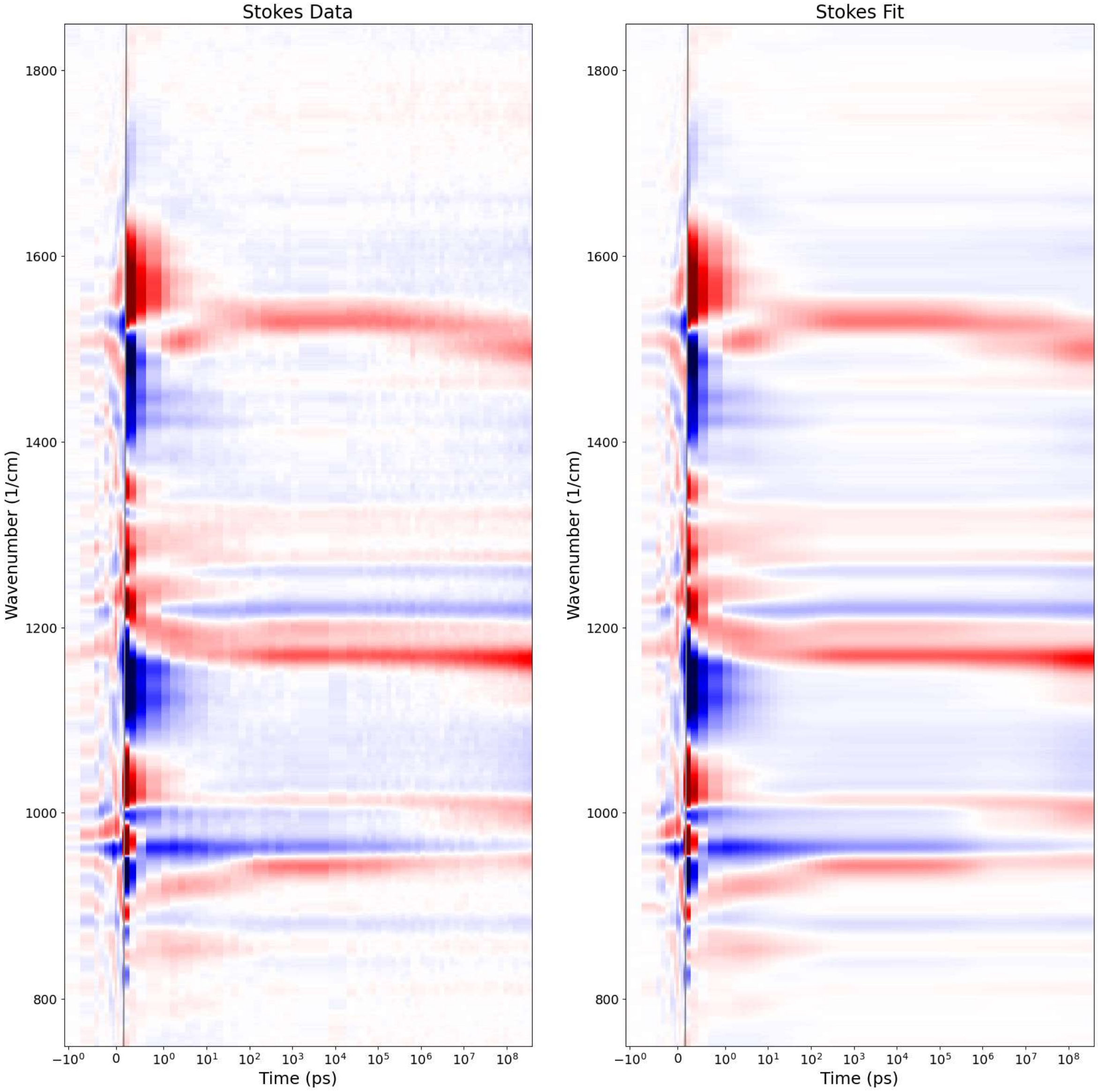

Fig. S5. Left panel: Heat map of the raw FSRS data of the *P. antarctica* P540/D661 mixture with actinic excitation at 520 nm. Right panel: fit result using the kinetic scheme shown in Fig. 4A for the P540 state with SADS shown in Fig. S4A and a sequential analysis of the D661 data with EADS shown in Fig. S4B.[1] Note that the time axis is linear until 1 ps, and logarithmic thereafter. The grey line indicates the time of the maximum of the IRF.

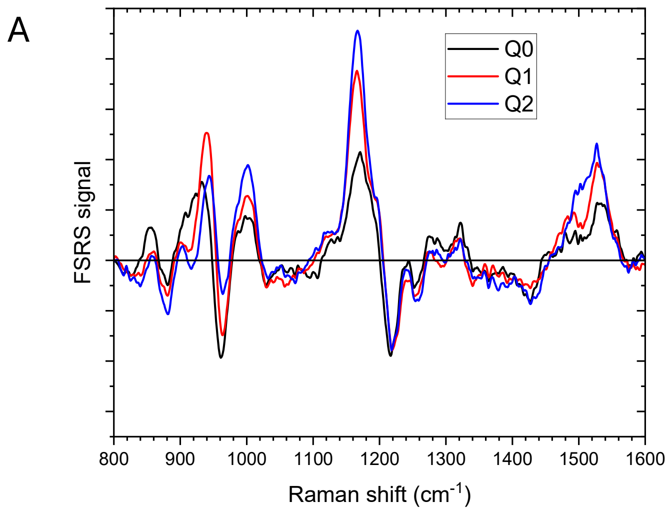

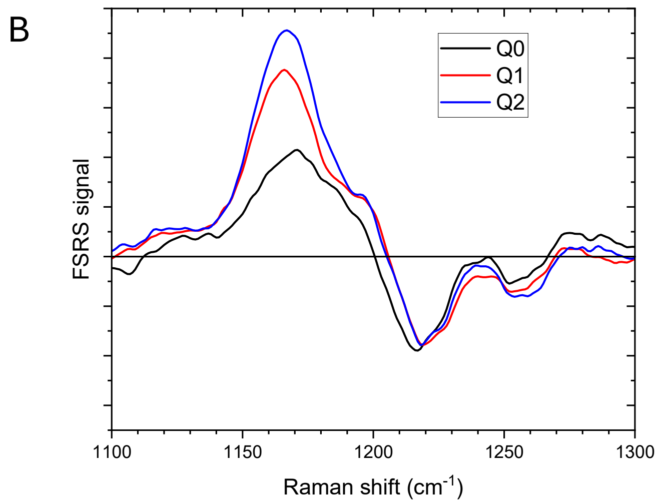

Fig. S6. (A) FSRS EADS of the *P. antarctica* bestrhodopsin P540 state, dataset II. (B) same as (A) zoomed in on the fingerprint region. The Q3/D661 intermediate was not detected because the time axis was terminated at 10 μs.

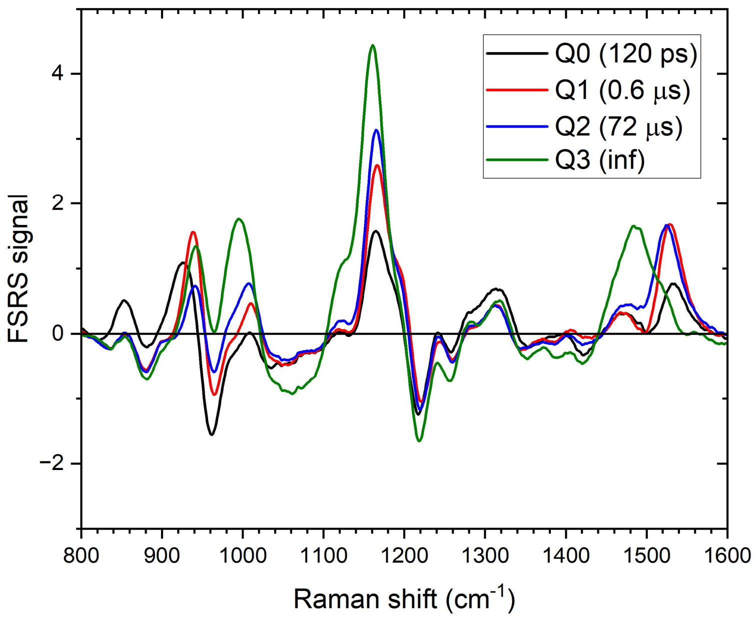

Fig. S7: Product state SADS for the *P. antarctica* bestrhodopsin Lysine mutant (K332A) P540 state upon actinic excitation at 515 nm, using the kinetic scheme shown in Fig. 4A.

(1) Kaziannis, S.; Broser, M.; Stokkum, I.; Dostal, J.; Busse, W.; Munhoven, A.; Bernardo, C.; Kloz, M.; Hegemann, P.; Kennis, J. T. M. Multiple retinal isomerizations during the early phase of the bestrhodopsin photoreaction. *Proceedings of the National Academy of Sciences of the United States of America* **2024**, *121* (12). DOI: 10.1073/pnas.2318996121.
